# Non-contact (touchless) monitoring of respiratory rate in a challenging anesthesia setting using a depth camera

**DOI:** 10.1007/s10877-025-01319-6

**Published:** 2025-07-10

**Authors:** David B. MacLeod, Philip Smit, André Antunes, Dean Montgomery, Paul S. Addison

**Affiliations:** 1 Department of Anesthesiology, Duke University Medical Center, Durham, NC 27710 US; 2 Medtronic Acute Care & Monitoring, Technopole Centre, Edinburgh, UK

**Keywords:** Respiratory disease, Respiratory rate, Anesthesia, Non-contact monitoring, Depth sensing camera

## Abstract

**Aim:**

We have developed a non-contact (“touchless”) system based on depth-sensing camera technology for continuous monitoring of respiratory activity. Previous work from our group has demonstrated high accuracy of the system in monitoring a wide range of respiratory rates and signal morphologies across diverse conditions, including variations in lighting, posture, and coverings. Here, we report on the system’s performance in a significantly more challenging anesthesia environment which included a wide range of respiratory rates and respiratory patterns, spontaneous and hand ventilated breathing, patient motion and caregiver interactions in the scene, and, in some cases, the presence of warming blankets covering the torso.

**Methods:**

Data was collected opportunistically from 34 healthy volunteers from two separate studies, both of which had the primary objective of investigating the relationship between depth of anesthesia monitoring and anesthetic agents (inhaled and intravenous) across a wide range of anesthetic concentrations and hypnotic states. Depth-sensing information was acquired using an Intel D415 RealSense™ camera and processed to extract frame-by-frame depth changes within the subject’s torso region corresponding to respiratory activity. A respiratory rate (RR_depth_) was calculated and output once-per-second from the device. This was compared to a combined reference (RR_ref_) derived from both a capnograph and an impedance-based respiratory monitor. Three time periods were evaluated: pre-anesthesia, intra-anesthesia and post-anesthesia.

**Results:**

The overall RMSD accuracy [bias] obtained for the combined data set was 1.92 [0.30] breaths/min. The performance results stratified according to pre-, intra-, and post-anesthesia stages were 1.71 [0.15], 1.95 [0.39] and 2.13 [0.08] breaths/min, respectively.

**Conclusions:**

We have demonstrated the ability to continuously track respiratory rate during challenging conditions within an anesthesia setting using our non-contact, touchless, monitoring technology. We believe that our findings support the potential utility for continuous non-contact monitoring of respiration in clinical areas, such as the post-anesthesia care environment.

**Supplementary Information:**

The online version contains supplementary material available at 10.1007/s10877-025-01319-6.

## Introduction

Accurate and routine monitoring of vital signs by nursing staff in the Post-Anesthesia Care Unit (PACU) is crucial to preventing postoperative complications. If complications occur, timely recognition and management are essential for a successful resolution [1]. It is widely acknowledged that inconsistent monitoring of vital signs and a lack of awareness regarding the importance of the physiological changes experienced by patients may contribute to a failure in detecting clinical deterioration. Therefore, vital signs observations offer crucial insights into a patient’s clinical status [2]. As respiratory dysfunction frequently occurs after general anesthesia, especially during early recovery, due to perioperative medications and mechanical ventilation, consistent and accurate assessments of respiratory function in the post-anesthesia setting is a critical element in ensuring patient safety and effective postoperative care. During early recovery, there is a risk of serious respiratory complications including hypoxemia, hypoventilation, airway obstruction, and residual neuromuscular blockade, which can lead to respiratory failure.

Respiratory rate is known to be an indicator of life-threatening conditions where a change in rate is often an early sign of a major complication. It has been reported that even a small change in respiratory rate of approximately four breaths/min, can indicate signs of serious clinical deterioration [3]. As such, respiratory rate is included in most early warning scoring systems designed to detect clinical deterioration [4, 5]. This enables a timely response to prevent further clinical decline, as changes in vital signs often precede significant patient deterioration. Through prompt and effective interventions, adverse patient outcomes can be minimized [6]. However, intermittent vital sign monitoring can be inaccurate, incomplete, and miss changes in physiological parameters that occur between monitoring, especially during times of understaffing [7, 8]. Respiratory rate specifically has been noted to be frequently missed and/or reported inaccurately when measured through traditional intermittent monitoring methods [9].

Depth sensing has emerged as a powerful new non-contact or ‘touchless’ modality for monitoring respiratory activity. As no attached sensor or trailing wire is required, the technology reduces patient restrictions and infection risks by eliminating physical sensors. Depth cameras capture the distance (depth) of every point in the camera’s field of view (in contrast with standard (i.e. RGB) cameras which sense colors). Depth-based monitoring systems are capable of accurately tracking small, localized changes in depth, including the rise and fall of the chest during inhalation and exhalation, thereby allowing for real-time monitoring of respiratory activity. A particular focus of recent research in the space has been around respiratory rate [10–14]. In our own work we have developed a depth sensing respiratory monitoring system which provides a range of respiratory information including 1) real-time visualization of breathing, 2) breathing waveform, 3) respiratory rate, and 4) relative measure of tidal volume [15]. We have demonstrated the accuracy of the system, within 1 breath/min, across a range of respiratory rates (4–40 breaths/min) [16]. The technology can monitor respiratory activity through coverings [17] and track respiratory rate accurately through rapid rate changes [18]. It has also been shown to track respiratory patterns induced by desaturations during a controlled hypoxia study [19] and accurately reflect respiratory disturbances in sleep apnea patients [20]. In addition, we recently reported on its use in monitoring inhalation: exhalation ratio, a useful marker of disease state in spontaneously breathing patients [21].

Here, we report on the performance of our depth-sensing camera system for the continuous non-contact monitoring of respiratory rate in a controlled anesthesia environment. This environment is significantly more challenging than in our previous studies as it includes a wide range of respiratory rates and respiratory patterns, spontaneous and hand ventilated breathing, patient motion and caregiver interactions in the scene, and, in some cases, the presence of warming blankets covering the torso.

## Methods

### Patient cohort

Adult volunteers were recruited opportunistically from two clinical studies which had the primary objective of investigating the relationship between depth of anesthesia and anesthetics across a wide range of anesthetic concentration and hypnotic states. The data from a total of 34 patients were collected for analysis (Study 1: *N* = 16, clinicaltrials.gov: NCT04466384. Study 2: *N* = 18, clinicaltrials.gov: NCT04602546). The studies offered a unique opportunity to measure respiratory rate across a wide range of anesthetic levels. In both studies participants underwent a protocol where anesthetic agents were administered to reach pre-defined concentration plateaus in a stepwise format. Study 1 involved the administration of total intravenous anesthesia, separated into two arms: (1) Propofol and (2) Propofol with Remifentanil. Study 2 involved the administration of inhaled volatile agents and had five arms: (1) Sevoflurane, (2) Sevoflurane with Remifentanil, (3) Sevoflurane with Fentanyl, (4) Desflurane, (5) Isoflurane. The studies were conducted in accordance with the Declaration of Helsinki and all local regulatory requirements. The studies were sponsored and funded by Medtronic. Both protocols were approved by the Institutional Review Board (IRB) at Duke University. Written informed consent was obtained for all participants prior to study procedures.

### Data acquisition

**Fig. 1:**
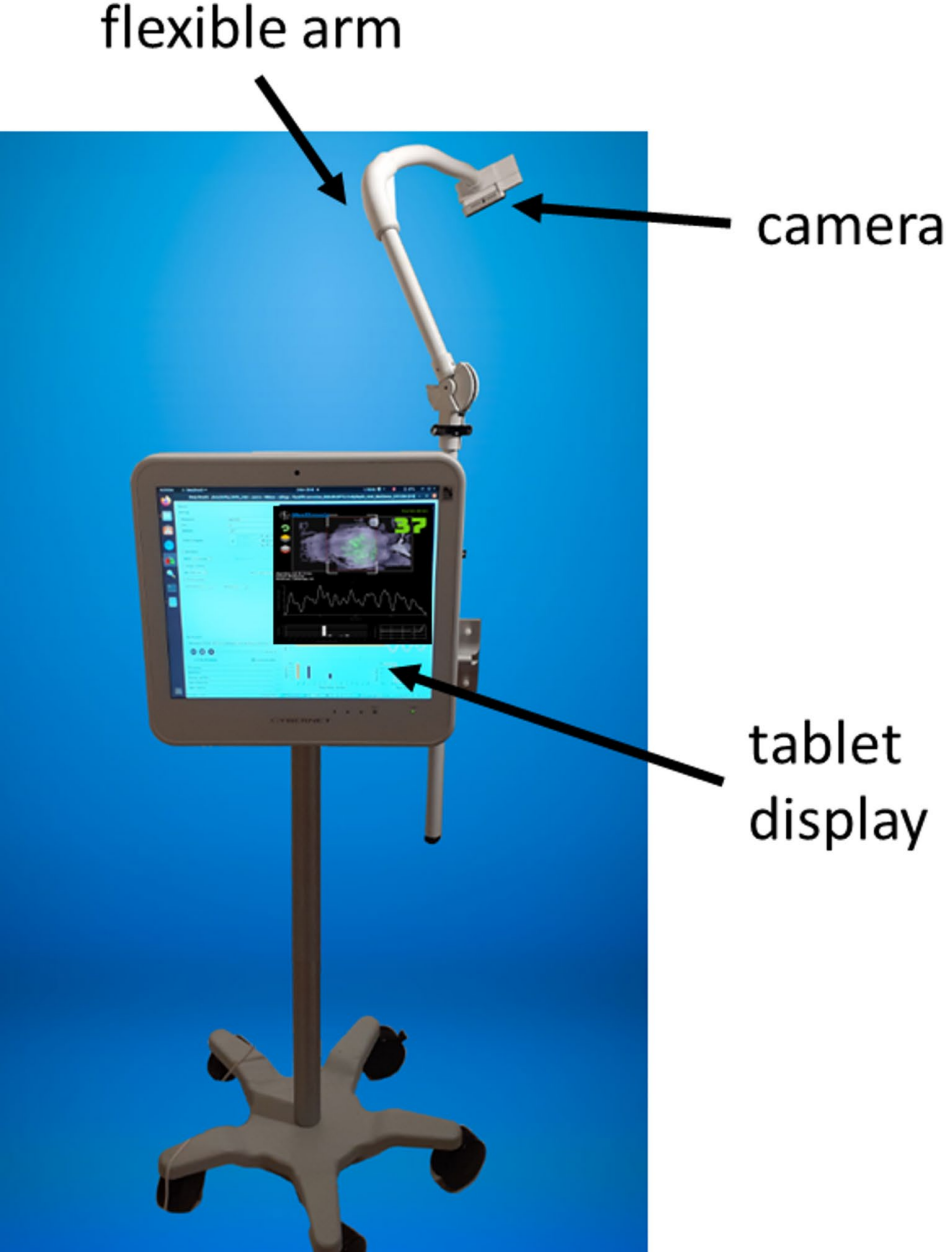
Touchless Monitoring Prototype Device

**Fig. 2:**
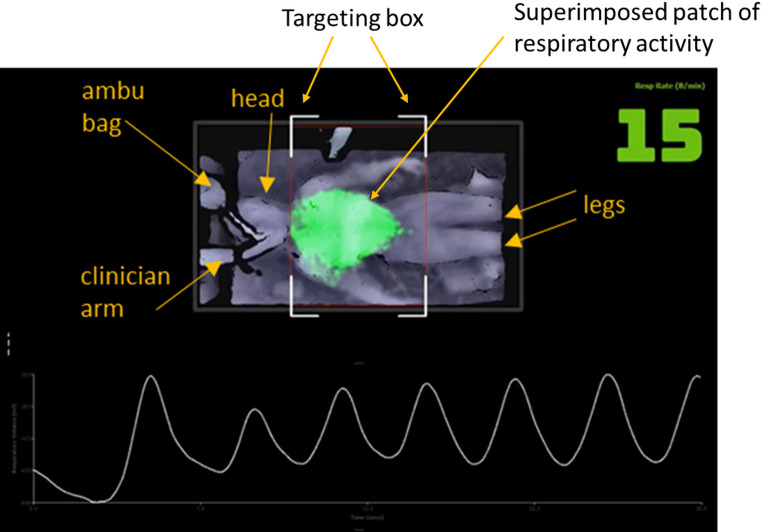
Touchless Monitoring Display from one of the Study Captures

Depth data was acquired from the scene using a prototype non-contact monitoring system. This incorporated a depth camera (RealSense™ D415, Intel, Santa Clara, CA) attached to a flexible arm and positioned at approximately 1.1 m above the patient’s torso. The acquisition system is shown in Fig. 1. Depth data was acquired from the scene at 15 frames per second (fps) and streamed to a tablet PC for processing in real-time using an in-house application written in C++. Respiratory activity manifests itself as a change in distances from the camera to the surface of the patient’s torso over time. The depth system generates a visible patch on the torso where respiratory activity is detected. Patch intensity is directly related to respiratory-induced changes in depth on the torso causing it to vary across the breathing cycle, thus providing a useful temporal visualization of breathing. A screenshot of the display is shown in Fig. 2. The frame-by-frame depth changes within the patch on the torso region are integrated over the patch to produce an incremental change in volume per frame. These are then integrated over time to produce a respiratory signal. This appears at the bottom of the display in Fig. 2. A respiratory rate, RR_depth_, is calculated from the respiratory signal and output once-per-second from the device. The system requires no calibration and the touchless respiratory signal output from the device was used directly in the analysis described herein with no further post-processing. A targeting box is integrated into the display to make it easier to position the camera above the subject in the correct location, allowing for consistent quality in the captured data. This is indicated by the four white-line box corners that can be seen in both images of Fig. 2. An example video clip from the touchless monitoring system is provided as Supplementary Material.

Reference respiratory rates were captured from both a *Capnostream™ 35* capnograph (Medtronic, Boulder, CO), (RR_cap_) and an *Exspiron™ 1Xi* non-invasive respiratory volume monitor (Respiratory Motion Inc., Waltham, MA), (RR_mon_) during the study. These reference respiratory rate signals were synchronized and resampled to 1 Hz for analysis purposes.

**Fig. 3:**
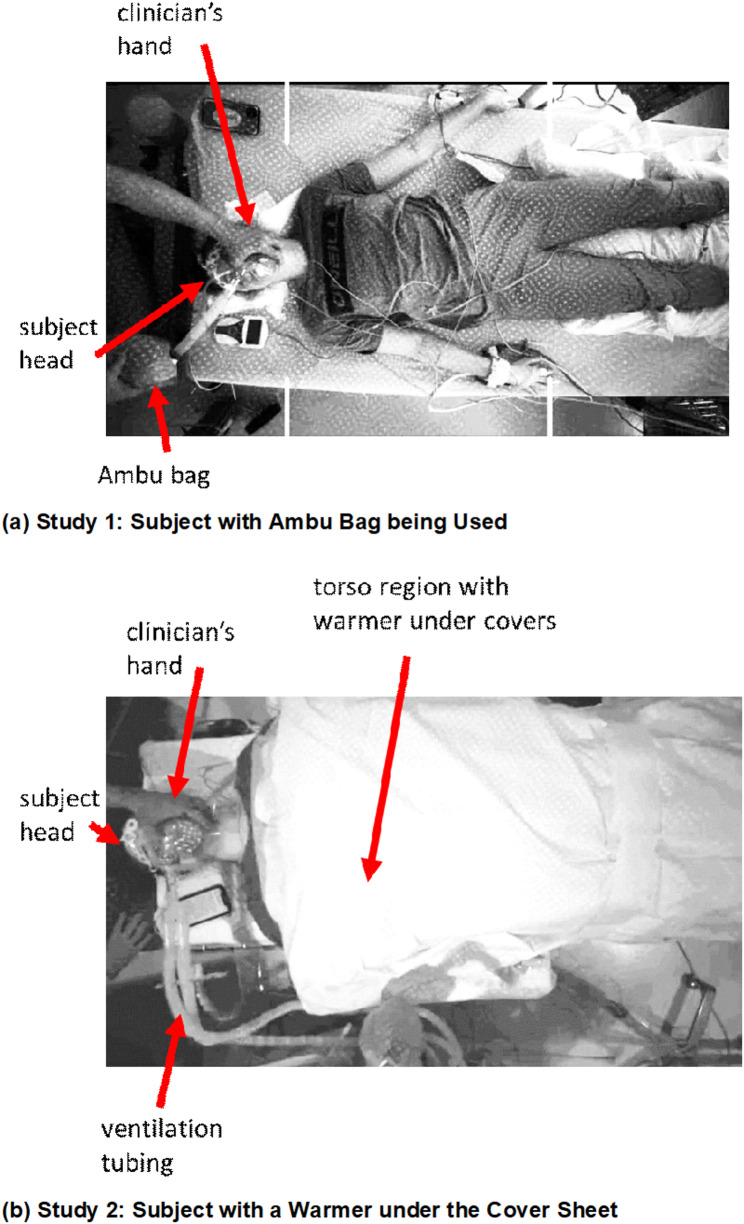
Example Images of Volunteers during the Trials

Manual ventilation with Ambu bag was used regularly in Study 1 to support respiration and could often be seen in the scene. Warmers were used for 16 participants in Study 2. Figure 3 contains examples of images taken from each study, where an Ambu bag is shown in Fig. 3(a) and a warmer in Fig. 3(b).

### Data analysis methodology

**Fig. 4:**
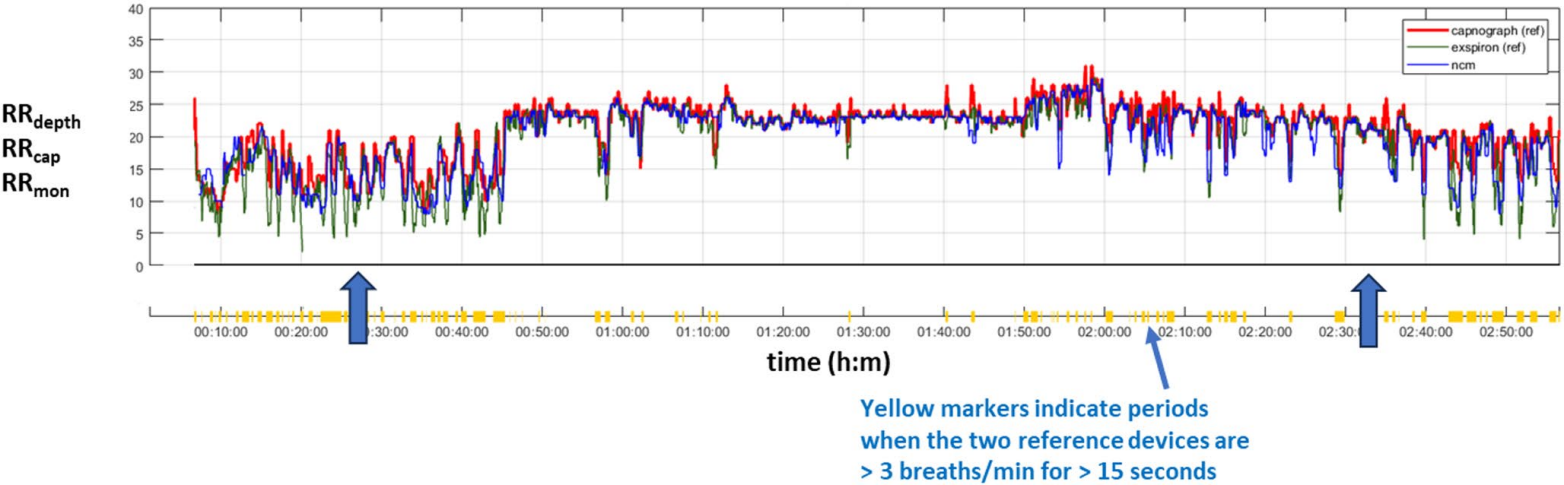
An example time series of RR _depth_, RR_cap,_ RR_mon_. The vertical arrows indicate the transition between pre, intra and post-anesthesia. RR_depth_ = blue line; RR_cap_ = red line; RR_mon_ = green line

Due to the excessive motion noise in the anesthesia environment, which can lead to erroneous and divergent reported rates from each device, we analyzed the performance of RR_depth_ only during periods where the two reference devices reported respiratory rates within 3 breaths/min of each other for at least 15 consecutive seconds. These we considered to be relatively stable reporting periods during which RR_depth_ was compared to a reference value of respiratory rate (RR_ref_) calculated as the mean value of RR_cap_ and RR_mon_. An example of the reported respiratory rates for the touchless system and the two reference devices for volunteers is provided in Fig. 4.

We analyzed the data as a whole and then independently as two protocol subgroups. We further split the analysis sets into three anesthesia stages: pre-anesthesia, intra-anesthesia and post-anesthesia. For Study 1, the start of intra-anesthesia was defined at the initiation of the intravenous anesthetic infusion. The transition point from intra-anesthesia to post-anesthesia was when the propofol was reduced from 1.5 to 0.5 µg/mL. For Study 2, the start of intra-anesthesia was defined at the initiation of the inhaled volatile anesthetic drug administration, and the end was defined as the point at which the volatile agent was switched off. These stages are marked in Fig. 4 by vertical arrows.

We employed linear regression to perform a comparative analysis of the resulting respiratory rates from the depth camera and the reference where the line of best fit and Pearson’s correlation (R) were calculated. Performance metrics in the form of bias and accuracy statistics were derived to compare the depth-derived respiratory rate, RR_depth_, with the reference RR_ref_. These are, respectively, the mean difference (bias) and the root mean squared difference (RMSD accuracy) between the test and reference values. That is:

$$\:bias=\frac{\sum\:_{i=1}^{N}\left({RR}_{depth}\left(i\right)-{RR}_{ref}\left(i\right)\right)}{N}\:\:\:\:\:\:\:\:\:\:\:\:\:\:\:\:\:\:\:\:\:\:\:\:\:\:\:\:\:\:\:\:\:\:\:\:\:\:\:\:\:\:\:\:\:\:\:\:\:\:\:\:\:\:\left[1\right]$$

and

$$\begin{array}{l}\:RMSD\:accuracy=\sqrt{\frac{\sum\:_{i=1}^{N}{\left({RR}_{depth}\left(i\right)-R{R}_{ref}\left(i\right)\right)}^{2}}{N}}\:\:\:\:\:\:\:\:\:\:\:\:\:\:\:\:\:\:\:\:\:\:\:\:\:\:\:\:\:\:\:\:\:\:\:\:\:\:\:\:\:\:\:\:\:\:\:\:\:\:\:\:\:\:\:\:\:\:\left[2\right]\end{array}$$

where “i” corresponds to the index of each of the RR pairs. A box plot of the resulting intra-patient RMSDs was also computed. Matlab™ (R2024a; Natick, Massachusetts) was used to perform the statistical analysis.

## Results

A total of 81.9 h of data was collected across both studies, with a mean duration of 144.5 min per subject. The ranges of recorded respiratory rates on the devices were: RR_depth_: 2 to 40; RR_cap_: 2 to 83; and RR_mon_: 2 to 47 breaths/min.

**Fig. 5:**
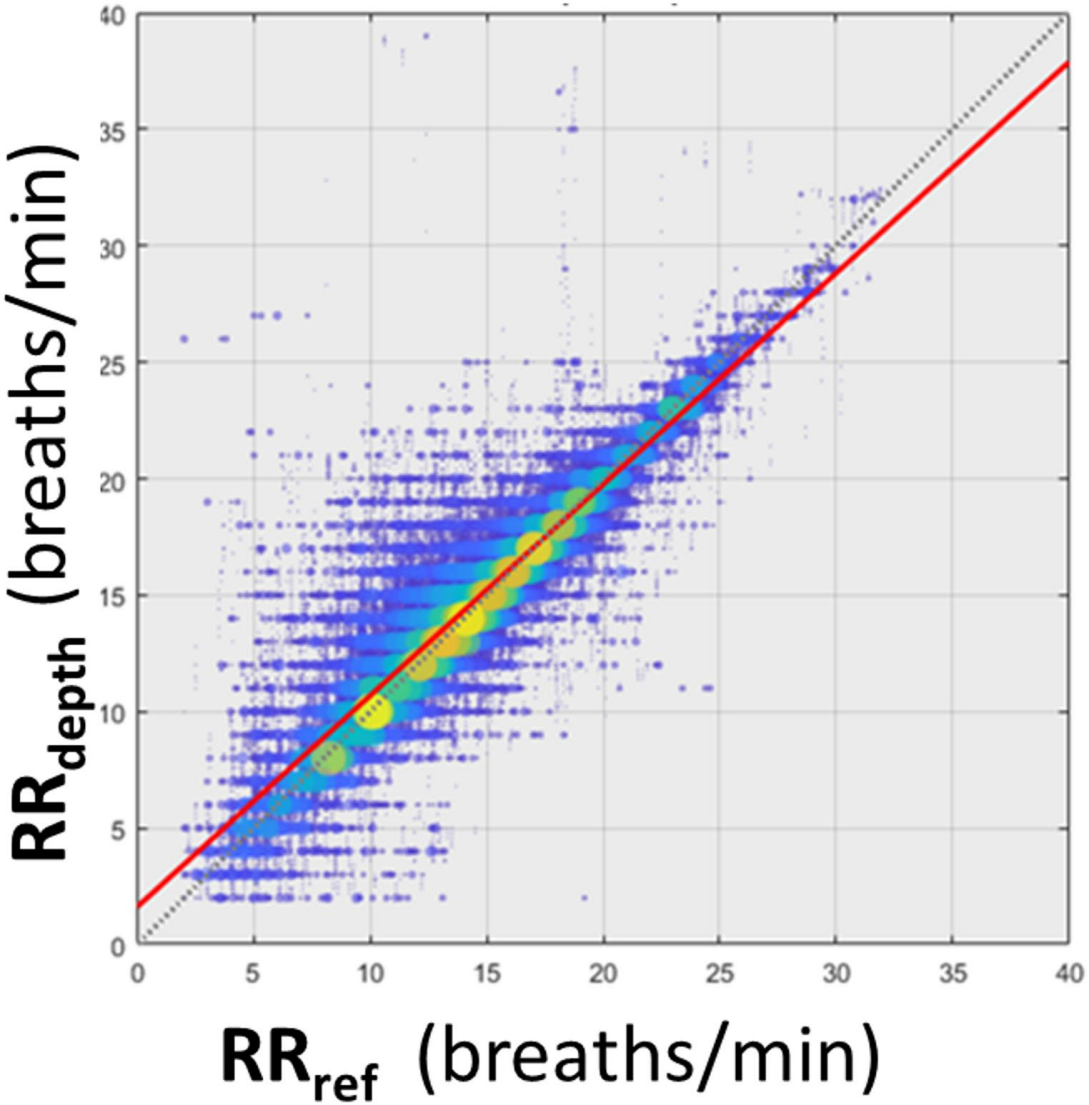
Scatter Plot of RR_depth_ against RR_ref_ for the Combined Data Set from Both Sites (All Data). The colors (blue through yellow) and the increasing size of discs indicate the increasing density of points at a location in the plot

The overall RMSD accuracy [bias] obtained for the combined data sets was 1.92 [0.30] breaths/min. The corresponding regression plot is shown in Fig. 5.

**Fig. 6:**
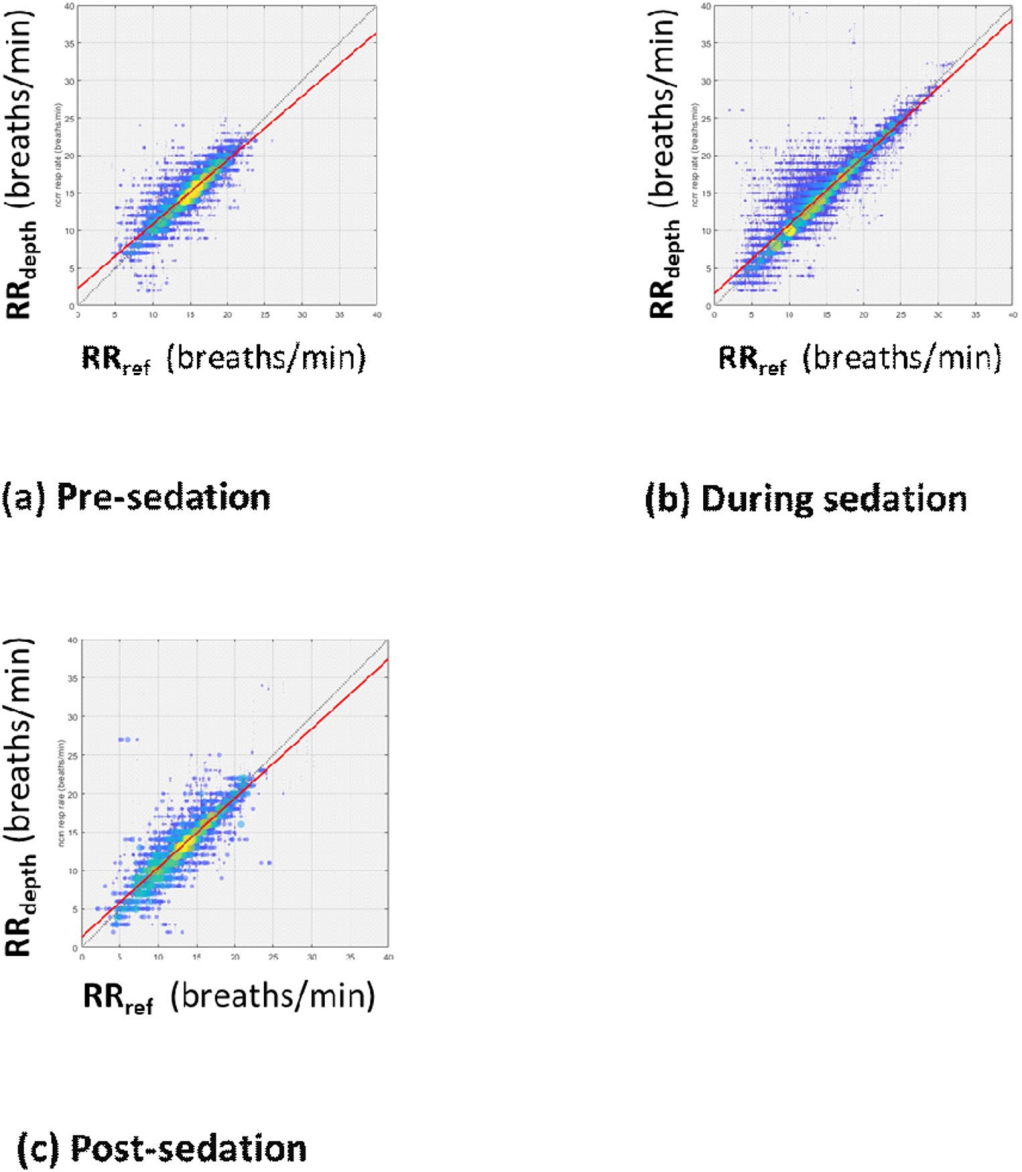
Scatter Plots of RR_depth_ against RR_ref_ for the Data split according to Anesthesia Period

The performance results stratified according to pre, intra and post anesthesia stages are presented in Figs. 6(a) to (c) respectively. The corresponding RMSD [bias] results were 1.71 [0.15], 1.95 [0.39] and 2.13 [0.08] breaths/min respectively.

**Fig. 7:**
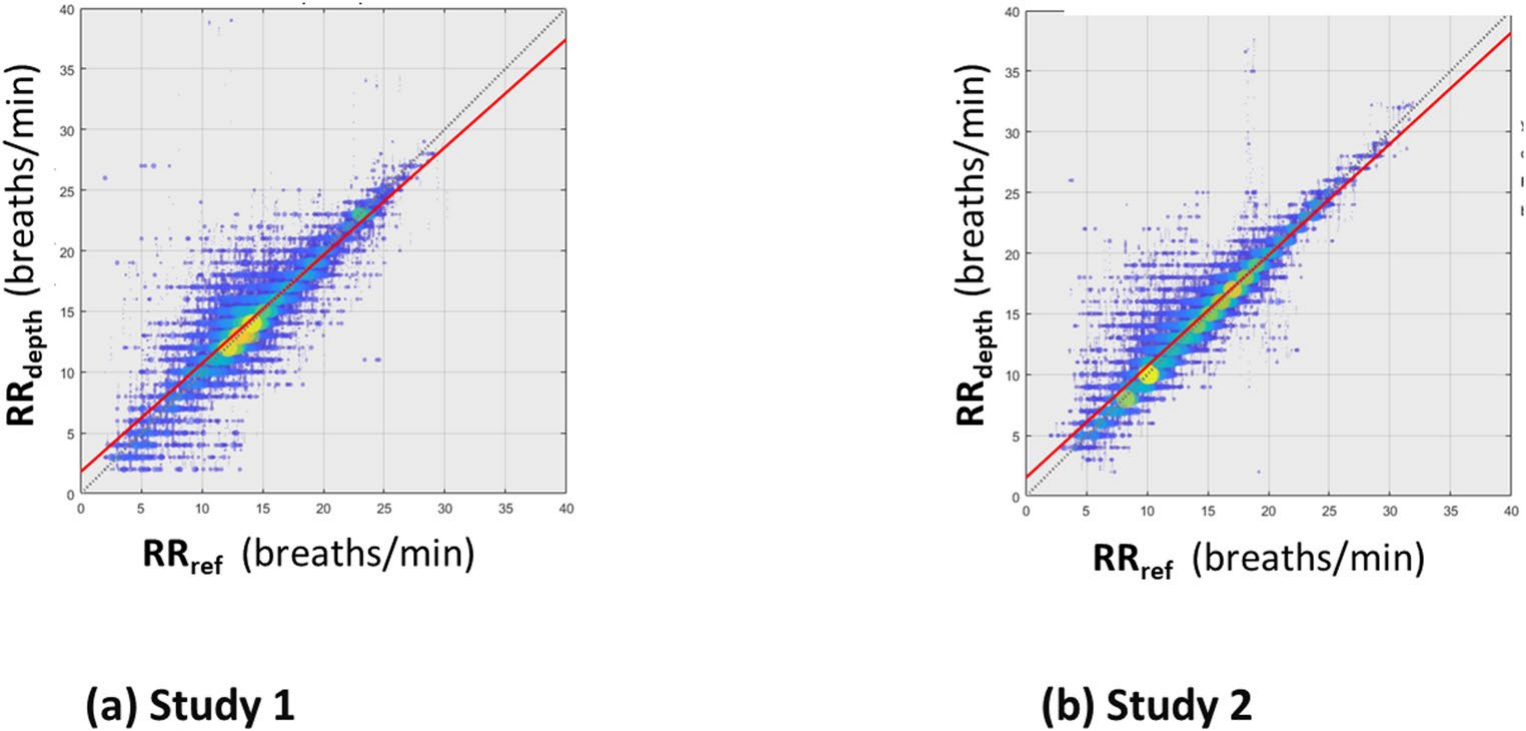
Scatter Plots of RR_depth_ against RR_ref_ for the Data split according to Study

The performance results stratified according to study are presented in Figs. 7(a) and 7(b) respectively. The corresponding RMSD [bias] results for Study 1 and Study 2 were 2.16 [0.25] and 1.75 [0.33] breaths/min respectively.

**Fig. 8:**
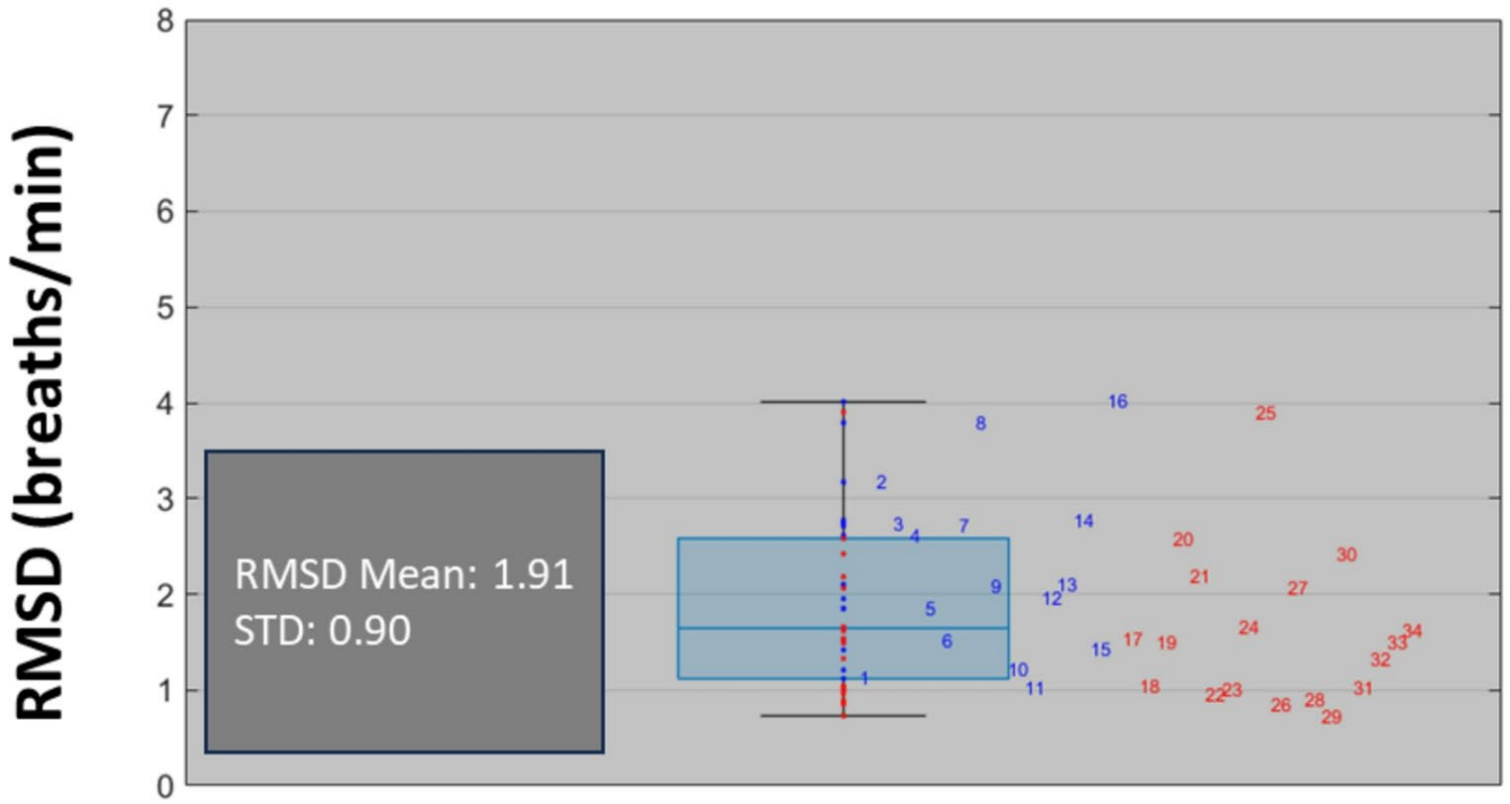
The Calculated RMSD Accuracies on a Per-Subject Basis. The individual subject data points are shown. Study 1 = blue. Study 2 = red

The RMSD accuracies calculated on a per-subject basis are plotted in Fig. 8.

## Discussion

We have developed a continuous non-contact monitoring technology for respiratory rate based on a depth camera system. This was tested in a challenging anesthesia environment comprising 34 patients undergoing various anesthesia regimens where an overall accuracy of 1.92 breaths/min was achieved. Sub-stratification according to both study and anesthesia stage found similar performances (range 1.71 to 2.16 breaths/min). These results all fit well within our target performance for the depth sensing respiratory rate technology of 3 breaths/minute accuracy across a range of respiratory rates from 4 to 40 breaths/min [16].

The study environment was extremely challenging as dynamic clinical interactions within the scene made for variable artefacts throughout data collection, all of which needed to be accounted for to obtain an accurate measurement of respiratory rate. One confounding factor included the constant interaction between clinician and participant, which caused considerable number of motion artefacts on the respiratory signal. Additionally, intermittent interference in the depth scene occurred due to the intermittent presence of the clinician and other moving equipment (e.g. the Ambu bag). As participants proceeded through the study protocol, they often exhibited a wide range of respiratory rates and irregular breathing patterns, which required careful analysis; and these could be generated through spontaneous breathing or ventilatory support (via the Ambu bag). In addition, warming blankets covered the torso of many of the volunteers. In light of these challenges, the results can be viewed as strong evidence that the technology performs accurately within difficult clinical environments.

There are a number of limitations to this study. The study participants were healthy volunteers undergoing a defined anesthesia study protocol, and not patients undergoing anesthesia in the clinical setting. Further studies in spontaneously breathing patients in the anesthesia setting, exhibiting a range of respiratory conditions, are needed to further understand and refine this technology for use in clinical practice. It would, for example, be interesting to track patients exhibiting respiratory compromise en route to respiratory failure in the PACU. The prototype system uses a trolley-based set-up with an arm that extended over the bed. For a system that could ultimately be deployed in a hospital environment, various design factors would have to be considered including a form factor that allows the camera system to be unobtrusive to the clinical staff and patients. We are currently considering alternative strategies for this including floor, bed and ceiling/wall mounted options.

A particular strength of the study was that a relatively comprehensive data set was captured for analysis comprising a total of 81.9 h of respiratory data from 34 patients covering a wide range of respiratory rates. In addition, we did not exclude any data from the analysis as long as a viable RR_ref_ was present. Further, we defined this reference respiratory rate formed from two reference devices where we tolerated both a wide range in their reported rates comprising a 6 breaths/min relative range (+ 3 to -3) and a relatively short 15 s period of required agreement between them.

Our results suggest that touchless monitoring is viable as an alternative modality for determining respiratory rate in challenging anesthesia environments. The system uses an off-the-shelf depth camera (Intel D415 RealSense™) and requires no hardware changes, calibration, post-processing or elaborate set-up. The camera is simply placed above the torso and the depth system produces the respiratory rate in real time. The technology also has the advantages of not requiring attached sensors or trailing wires and reporting the vital sign as soon as the patient is present in the scene thus improving workflow and patient comfort. Such automated continuous monitoring could prevent gaps in the assessment and recording of vital signs in practice. A further advantage is in reducing the risk of cross-contamination and infection. The technology allows for real-time tracking of the patient’s status over time as they deteriorate and/or respond to therapy and, as such, could potentially be integrated with remote monitoring systems, allowing healthcare providers to monitor multiple patients simultaneously, improving the efficiency of care in the PACU. Outside the PACU, the technology also fits well with remote and non-contact monitoring of the patient at home: another challenging area for patient monitoring which gained significant traction during the global COVID pandemic [22, 23] as sicker patients were sent home but still required careful assessment of their respiratory status.

## Conclusion

The viability of continuous non-contact monitoring of respiratory rate has been demonstrated within a significantly challenging anesthesia setting. We believe that the technology may have particular utility in the PACU due to the likelihood of post-anesthesia respiratory complications that can be detected earlier with improved respiratory monitoring. Further studies will be required in this area of care to confirm this.

## Electronic supplementary material

Below is the link to the electronic supplementary material.

**Supplementary Material 1**: A video clip is attached as supplementary material showing the depth image video display when a volunteer was being provided with ventilatory assistance using an Ambu bag during the study. The Ambu bag is visible in the bottom left hand corner of the scene and the clinician’s hand and arm can be seen in the first few seconds of the video clip as they touch the subject.

## Data Availability

The source data contains identifiable video of the participant volunteers and cannot be shared in order to protect study participant privacy.
